# Characterization of Pancreatic Islets in Two Selectively Bred Mouse Lines with Different Susceptibilities to High-Fat Diet-Induced Glucose Intolerance

**DOI:** 10.1371/journal.pone.0084725

**Published:** 2014-01-13

**Authors:** Mototsugu Nagao, Akira Asai, Wataru Inaba, Momoyo Kawahara, Yuki Shuto, Shunsuke Kobayashi, Daisuke Sanoyama, Hitoshi Sugihara, Soroku Yagihashi, Shinichi Oikawa

**Affiliations:** 1 Department of Endocrinology, Diabetes and Metabolism, Graduate School of Medicine, Nippon Medical School, Tokyo, Japan; 2 Department of Pathology and Molecular Medicine, Hirosaki University Graduate School of Medicine, Hirosaki, Japan; College of Tropical Agriculture and Human Resources, University of Hawaii, United States of America

## Abstract

Hereditary predisposition to diet-induced type 2 diabetes has not yet been fully elucidated. We recently established 2 mouse lines with different susceptibilities (resistant and prone) to high-fat diet (HFD)-induced glucose intolerance by selective breeding (designated selectively bred diet-induced glucose intolerance-resistant [SDG-R] and -prone [SDG-P], respectively). To investigate the predisposition to HFD-induced glucose intolerance in pancreatic islets, we examined the islet morphological features and functions in these novel mouse lines. Male SDG-P and SDG-R mice were fed a HFD for 5 weeks. Before and after HFD feeding, glucose tolerance was evaluated by oral glucose tolerance test (OGTT). Morphometry and functional analyses of the pancreatic islets were also performed before and after the feeding period. Before HFD feeding, SDG-P mice showed modestly higher postchallenge blood glucose levels and lower insulin increments in OGTT than SDG-R mice. Although SDG-P mice showed greater β cell proliferation than SDG-R mice under HFD feeding, SDG-P mice developed overt glucose intolerance, whereas SDG-R mice maintained normal glucose tolerance. Regardless of whether it was before or after HFD feeding, the isolated islets from SDG-P mice showed impaired glucose- and KCl-stimulated insulin secretion relative to those from SDG-R mice; accordingly, the expression levels of the insulin secretion-related genes in SDG-P islets were significantly lower than those in SDG-R islets. These findings suggest that the innate predispositions in pancreatic islets may determine the susceptibility to diet-induced diabetes. SDG-R and SDG-P mice may therefore be useful polygenic animal models to study the gene–environment interactions in the development of type 2 diabetes.

## Introduction

Gene–environment interactions play a crucial role in the development of type 2 diabetes. For analyzing genetic factors, the polygenic background of selectively bred animal models has been investigated [1]. For instance, the Goto-Kakizaki (GK) rat [2] and Nagoya-Shibata-Yasuda (NSY) mouse [3] are non-obese diabetic models produced by repetitive selective breeding for impaired glucose metabolism. Through the analyses of these polygenic rodent models, increasing numbers of candidate genes for the pathogenesis of type 2 diabetes have been identified, most of which well resemble the genetic basis of type 2 diabetes in humans [1], [4], [5].

Contemporary environmental factors (*e.g*., nutritional excess and sedentary lifestyle) cause obesity, which leads to insulin resistance in peripheral tissue [6], [7]. However, not all obese individuals with insulin resistance develop type 2 diabetes because the functional and morphological compensation capacities of β cells against insulin resistance vary between individuals [8]. In rodent models, animals with high-fat diet (HFD)-induced obesity are chiefly used for assessing the impact of excess dietary fat as an environmental factor. However, the propensity for developing diet-induced diabetes varies widely even in a single strain [9]. Thus, individual differences in susceptibility to environmental factors are postulated to be determined by genetic factors. Existing polygenic models, which develop diabetes spontaneously [10], may therefore not be always appropriate to investigate the predisposition to lifestyle-related disorders because environmental factors had not been taken into account in their selective breeding.

To establish novel rodent models that can mimic the gene–environment interactions in the development of type 2 diabetes, we have performed a selective breeding of mice. In brief, using 3 inbred strains (C57BL/6, C3H, and AKR) as background, mice exhibiting superior and inferior glucose tolerance after HFD feeding have been bred repetitively to establish 2 distinct mouse lines with different susceptibilities (resistant and prone) to HFD-induced glucose intolerance, designated selectively bred diet-induced glucose intolerance-resistant (SDG-R) and -prone (SDG-P), respectively [11]. Given that SDG-P mice show evident glucose intolerance with mild obesity after HFD feeding as compared with SDG-R mice, these mice may serve as appropriate models for investigating hereditary predisposition to diet-induced diabetes. In this study, we examined pancreatic islet morphological features and functions in these novel mouse lines to investigate the predisposition to HFD-induced glucose intolerance in islets.

## Materials and Methods

### Animals

Male SDG-R and SDG-P mice (15^th^–17th generations [11]) bred at the Institute for Animal Reproduction (Kasumigaura, Japan) were used. The mice were housed at 3–5 animals per cage and maintained in a temperature-controlled room on a 14-h light/10-h dark cycle, with free access to water and standard rodent chow (MF, Oriental Yeast, Tokyo, Japan), unless otherwise specified. SDG-R and SDG-P mice were fed with HFD (Quick Fat, CLEA Japan, Tokyo), providing 32% energy as fat, for 5 weeks (5–10 weeks of age). Before (4–5 weeks of age) and after HFD feeding (10–11 weeks of age), oral glucose tolerance test (OGTT), insulin tolerance test (ITT), and morphometric and functional analyses of pancreatic islets were performed. This study was conducted under approval from the institutional animal care and use committee of Nippon Medical School.

### Oral Glucose Tolerance Test

After overnight-fasted blood glucose levels were measured, a 20% glucose solution (40 and 60 mg glucose per mouse before and after HFD feeding, respectively) was orally administered, and blood glucose levels were measured at 30, 60, and 120 min after the administration with a glucose sensor (Glutest Neo Super, Sanwa Kagaku Kenkyusho, Nagoya, Japan) by tail bleeding. To evaluate early-phase insulin response, blood plasma was prepared from the tail vein blood before and at 15 and 30 min after the glucose challenge at 5 weeks of age. Plasma insulin levels were measured using an ultrasensitive mouse insulin ELISA kit (Morinaga, Yokohama, Japan).

### Insulin Tolerance Test

ITT was performed in accordance with a recommendation from the Mouse Metabolic Phenotyping Center (MMPC) Consortium [12]. After 6-h-fasted blood glucose levels were measured, insulin (Humulin R, Eli Lilly Japan, Tokyo) was injected intraperitoneally at 0.5 U/kg of body weight. Blood glucose levels were measured at 15, 30, 60, and 90 min after the injection as described earlier.

### Morphometric Analysis of Islet Cells

The overnight-fasted mice were killed by blood withdrawal from the inferior vena cava under anesthesia. The pancreas was excised and fixed in neutral-buffered formalin and embedded in paraffin. After reviewing the sections (4-µm thickness) with hematoxylin-eosin staining, contiguous sections were doubly immunostained for glucagon and insulin to identify α and β cells, respectively [13], [14]. Quantitative evaluations of the islet areas and volume densities of the α and β cells were performed using a computer-assisted point-counting method on Axio Image A1 microscope (Carl Zeiss, Oberkochen, Germany) and Nikon DS-Fi1-L2 digital camera system (Nikon, Tokyo, Japan) with the Image J software (version 1.46c, Wayne Rasband, National Institutes of Health) [13]–[15]. The morphometric analysis was performed by examiners who were unaware of the assignment of the specimens.

### Islet Isolation and Insulin Secretion *in Vitro*

Pancreatic islets were isolated by pancreatic duct injection of collagenase solution (1 mg/mL in Krebs-Ringer bicarbonate buffer [KRB] containing 0.2% bovine serum albumin [BSA]), followed by digestion at 37°C for 15 min with gentle shaking. Islets were then picked up manually under a stereomicroscope. A group of 10 islets of similar size was transferred into a cell culture filter insert (12-µm pores; Millicell, EMD Millipore, Billerica, MA, USA) in a 24-well plate containing Roswell Park Memorial Institute 1640 medium (containing 5.5 mmol/l glucose and 10% fetal bovine serum) and incubated overnight [16]. After the insert with islets was rinsed twice for 15 min with 1.4 mmol/l glucose in KRB containing 0.2% BSA, the islets were incubated in 2.8 mmol/l glucose for 60 min (low glucose concentration). The islets were then rinsed with 1.4 mmol/l glucose and treated with 16.7 mmol/l glucose for 60 min (high glucose concentration), after which the islets were rinsed again and treated with 34.8 mmol/l KCl for 60 min (high KCl concentration). The solutions (low glucose, high glucose, and high KCl concentrations) were collected for insulin assay. To analyze the intracellular insulin content, the islets were sonicated in distilled water and suspended in an acid-ethanol solution.

### RNA Isolation and Quantitative RT-PCR

Total RNA was extracted from freshly isolated islets using the Isogen reagent (Nippon Gene, Tokyo, Japan), and cDNA was generated by PrimeScript RT reagents (Takara Bio, Otsu, Japan) according to the manufacturers' instructions. Gene expression was analyzed using the ABI 7500 Fast real-time polymerase chain reaction system with the use of commercial primers of TaqMan Gene Expression Assays (Applied Biosystems, Foster City, CA, USA). The differences in gene expression were calculated by the comparative ΔΔCT method of relative quantification (normalized to *Gapdh*).

### Statistical Analysis

Data were expressed as mean ± standard error of mean (SEM). Mean values were compared using the Student *t* test, and *p*<0.05 was considered statistically significant. Statistical analyses were performed using the JMP 9.0.2 software (SAS Institute, Cary, NC, USA).

## Results

### HFD-induced Hyperglycemia in SDG-P Mice

**Figure 1:**
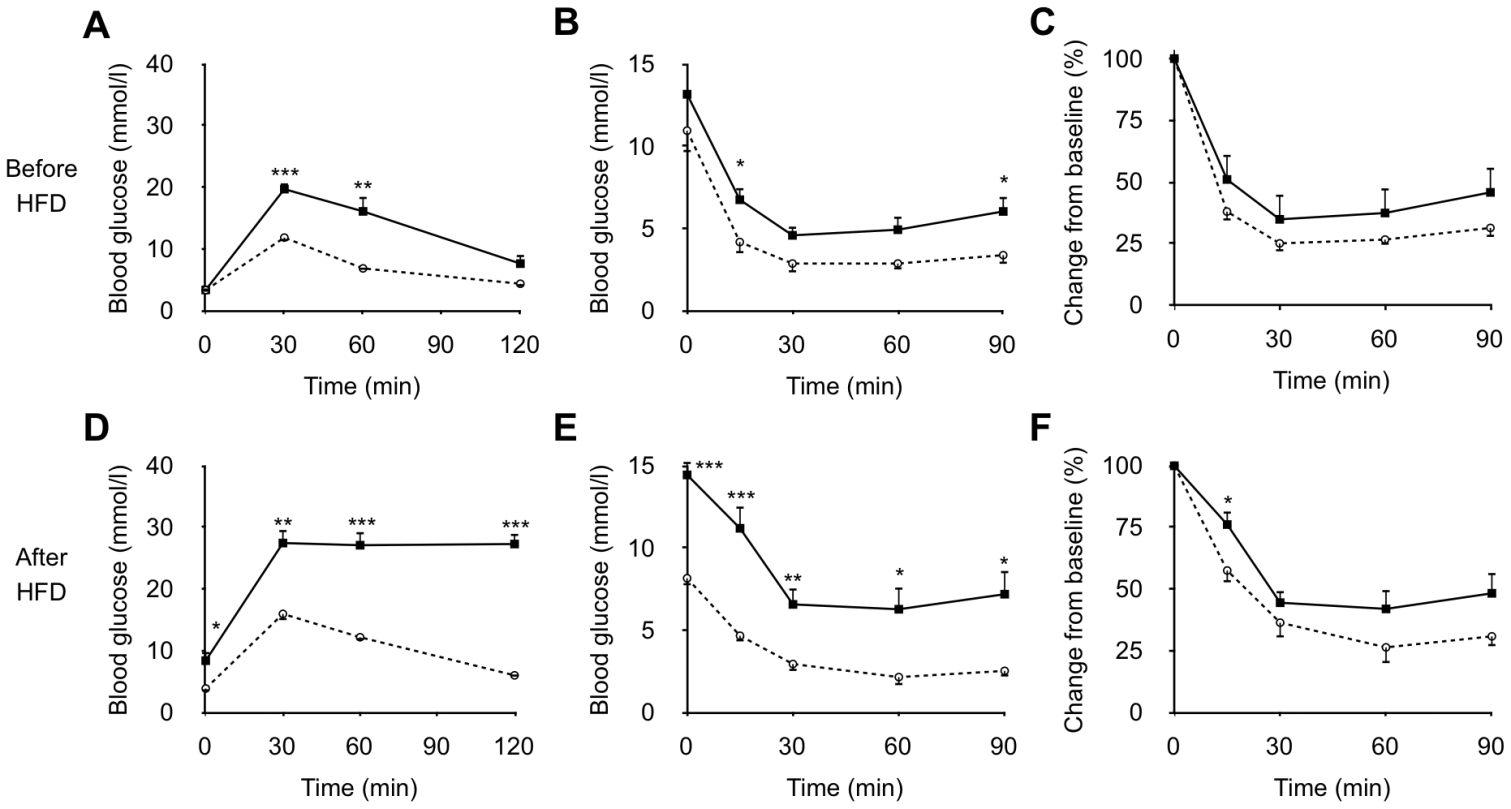
Glucose tolerance and insulin sensitivity in SDG-R and SDG-P mice. Blood glucose levels in OGTT before (**A**) and after HFD feeding (**D**). Blood glucose levels in ITT before (B) and after HFD feeding (E). Relative blood glucose levels from the baseline in ITT before (**C**) and after HFD feeding (**F**). Mean ± SEM (n = 5–6). SDG-R, open circle; SDG-P, closed square. *p<0.05, **p<0.01, ***p<0.001, *versus* SDG-R mice.

In OGTT, SDG-P mice showed modestly higher postchallenge blood glucose levels before HFD feeding (Figure 1A). The glucose intolerance in SDG-P mice became more evident after the 5-week HFD feeding (Figure 1D). In ITT, although blood glucose concentrations of SDG-P mice were higher than those of SDG-R at 15 and 90 min before HFD feeding (Figure 1B), the percent changes form baseline were not significantly different between the 2 lines of mice (Figure 1C). After HFD feeding, SDG-P mice showed evidently higher blood glucose concentrations in ITT (Figure 1E), and the 15-min value was significantly higher than that of SDG-R mice even when the values were expressed as percent changes form baseline (Figure 1F).

**Figure 2:**
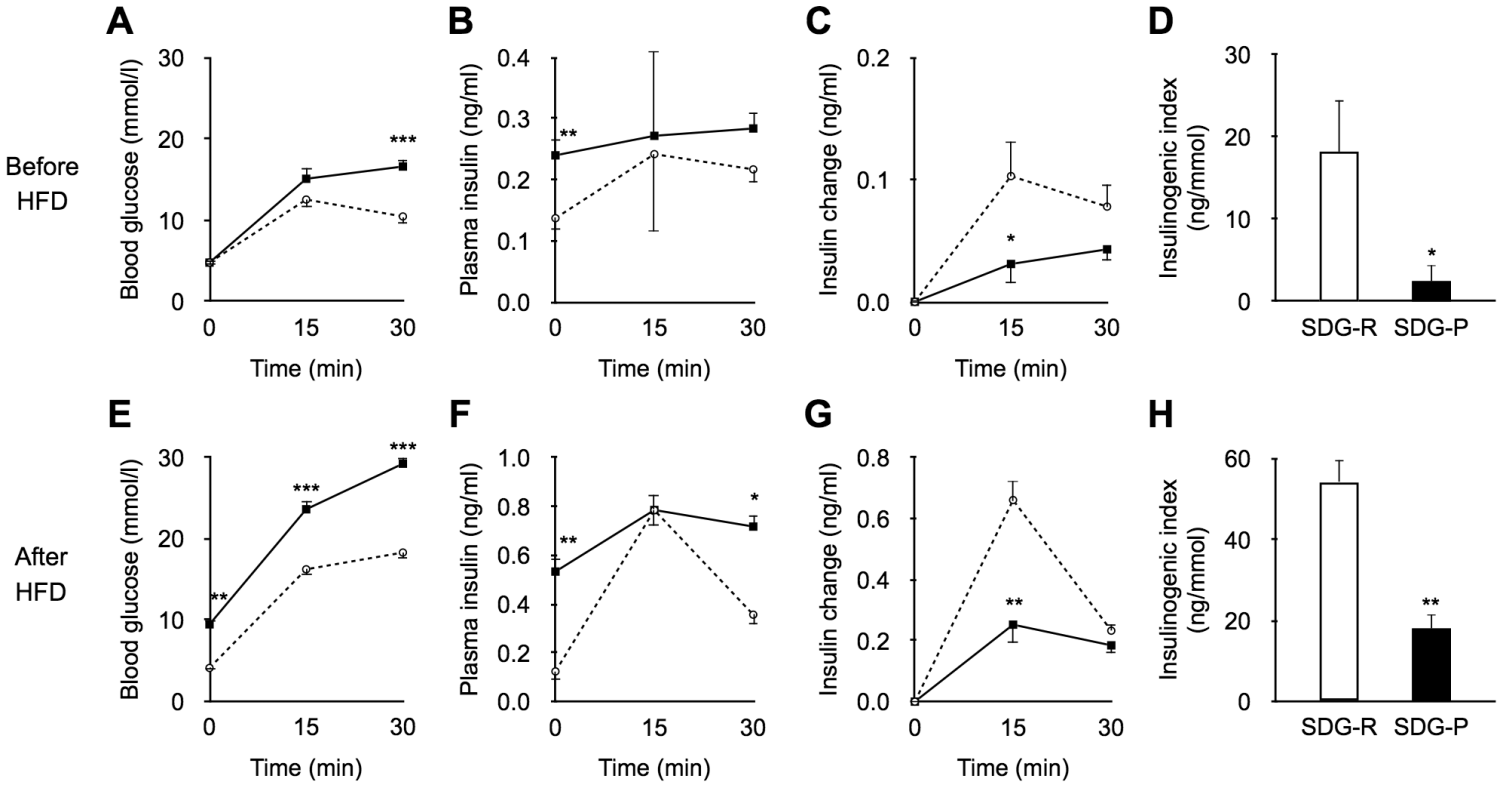
Glucose induced insulin secretion *in vivo*. Blood glucose and plasma insulin levels at 0, 15, and 30(**A–D**) and after HFD feeding (**E–H**). **A**, **E**; Blood glucose levels. **B**, **F**; Plasma insulin levels. **C**, **G**; Changes in plasma insulin levels from baseline. **D**, **H**; Insulinogenic index (Δ[insulin]_0–15 min_/Δ[glucose]_0–15 min_). Mean ± SEM (before HFD, n = 13–14; after HFD, n = 4–6). SDG-R, open circle/column; SDG-P, closed square/column. *p<0.05, **p<0.01, ***p<0.001, *versus* SDG-R mice.

No significant differences were observed in the post-glucose challenge insulin levels, whereas the fasting-state insulin levels in SDG-P mice were higher than those in SDG-R mice at 5 weeks of age (Figure 2B). Although no significant differences were observed in blood glucose and insulin levels at 15 min in OGTT (Figure 2A, B), the insulin response at 15 min (incremental insulin levels) in SDG-P mice was lower than that in SDG-R mice (Figure 2C). Consequently, the insulinogenic index (Δ[insulin]_0–15 min_/Δ[glucose]_0–15 min_) was significantly lower in SDG-P mice than in SDG-R mice (Figure 2D). The lower post-glucose challenge insulin response in SDG-P mice was also shown after HFD feeding (Figure 2E–H).

While no significant difference was observed in body weight between the 2 lines before HFD feeding (SDG-R mice, 15.2±0.9 g; SDG-P mice, 16.1±1.0 g: *p* = 0.53), SDG-P mice gained more body weight than SDG-R mice during the 5-week HFD feeding period (body weight after HFD feeding: SDG-R mice, 25.6±1.4 g; SDG-P mice, 32.8±1.7 g: *p* = 0.008).

### Morphological Analysis of Islets

**Figure 3:**
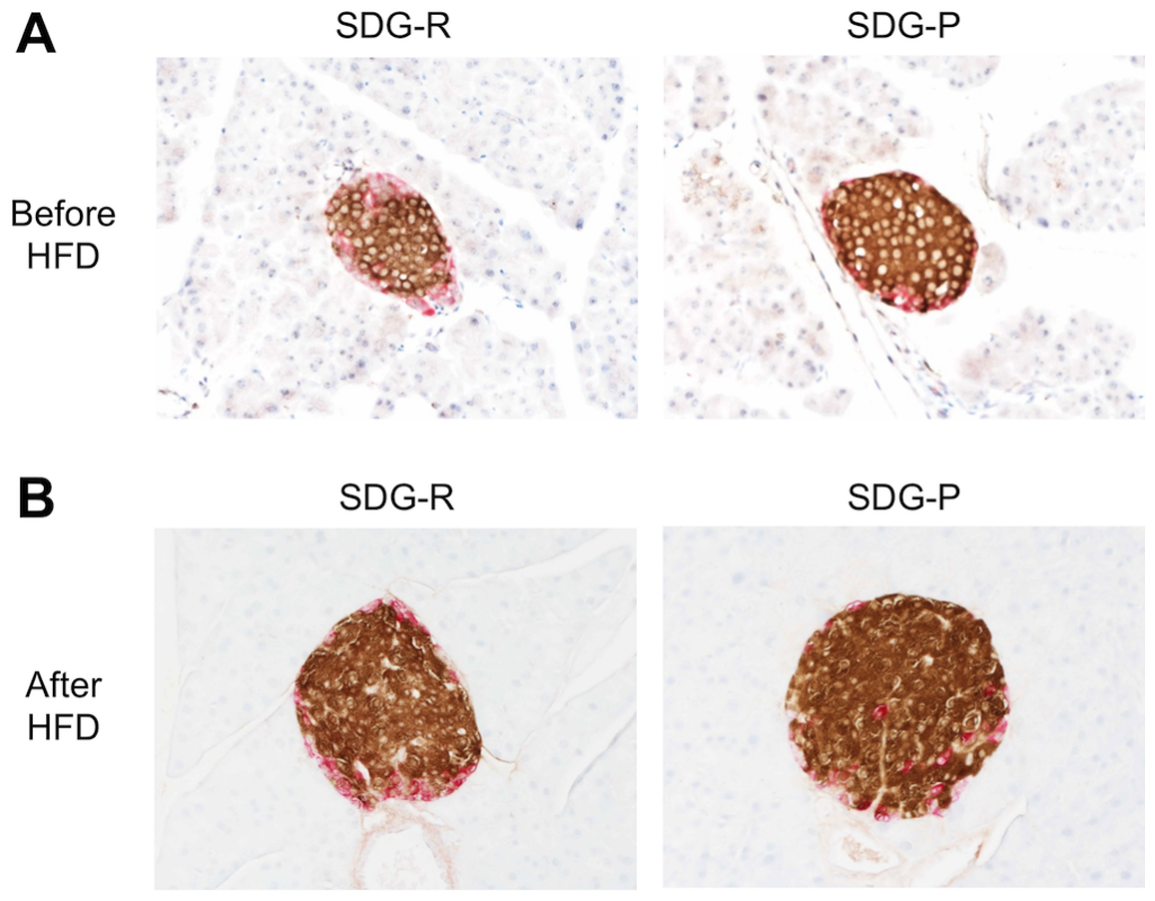
Immunostaining images of pancreatic islet in SDG-R and SDG-P mice. Immunostaining images of pancreatic islet before (**A**) and after 5-week HFD feeding (**B**). Double immunostaining for insulin (brown) and glucagon (red). Original magnification, ×200.

Before HFD feeding, no significant differences in pancreatic weight, islet density, and composition of islet endocrine cells were observed in the morphometric analysis between the 2 mouse lines (Figure 3, Table 1). In contrast, HFD-fed SDG-P mice showed greater pancreatic weight and 2-fold larger β cell mass than SDG-R mice. Although the α cell density in HFD-fed SDG-P mice was lower than that in SDG-R mice, no significant differences in α cell mass were observed between the 2 lines before and after HFD feeding. The HFD-feeding induced pancreatic weight gain and β cell proliferation in both lines of mice, but it did not affect α cell mass.

**Table 1:** Morphometric data of pancreatic islets in SDG-R and SDG-P mice.

	Pancreas	Islet	β Cell		α Cell	
	Weight (mg)	Density (%)	Density (%)	Mass (mg)	Density (%)	Mass (mg)
Before HFD^a^						
SDG-R	134±9	0.68±0.08	0.59±0.06	0.77±0.09	0.091±0.022	0.12±0.03
SDG-P	155±5	0.68±0.08	0.60±0.06	0.93±0.09	0.076±0.018	0.12±0.03
After HFD^b^						
SDG-R	207±9^†††^	0.92±0.08	0.85±0.08†	1.77±0.22^††^	0.072±0.011	0.15±0.02
SDG-P	302±28*,†††	1.12±0.15†	1.08±0.15†	3.52±0.70^**,††^	0.039±0.004^**^	0.13±0.02

Mean ± SEM (n = 5–6).

^a^ Five weeks of age.

^b^ Ten weeks of age.

*p<0.05, **p<0.01, *versus* SDG-R mice at the same age.

†*p*<0.05, ††*p*<0.01, †††*p*<0.01, *versus* before HFD feeding in the same line of mice.

**Figure 4:**
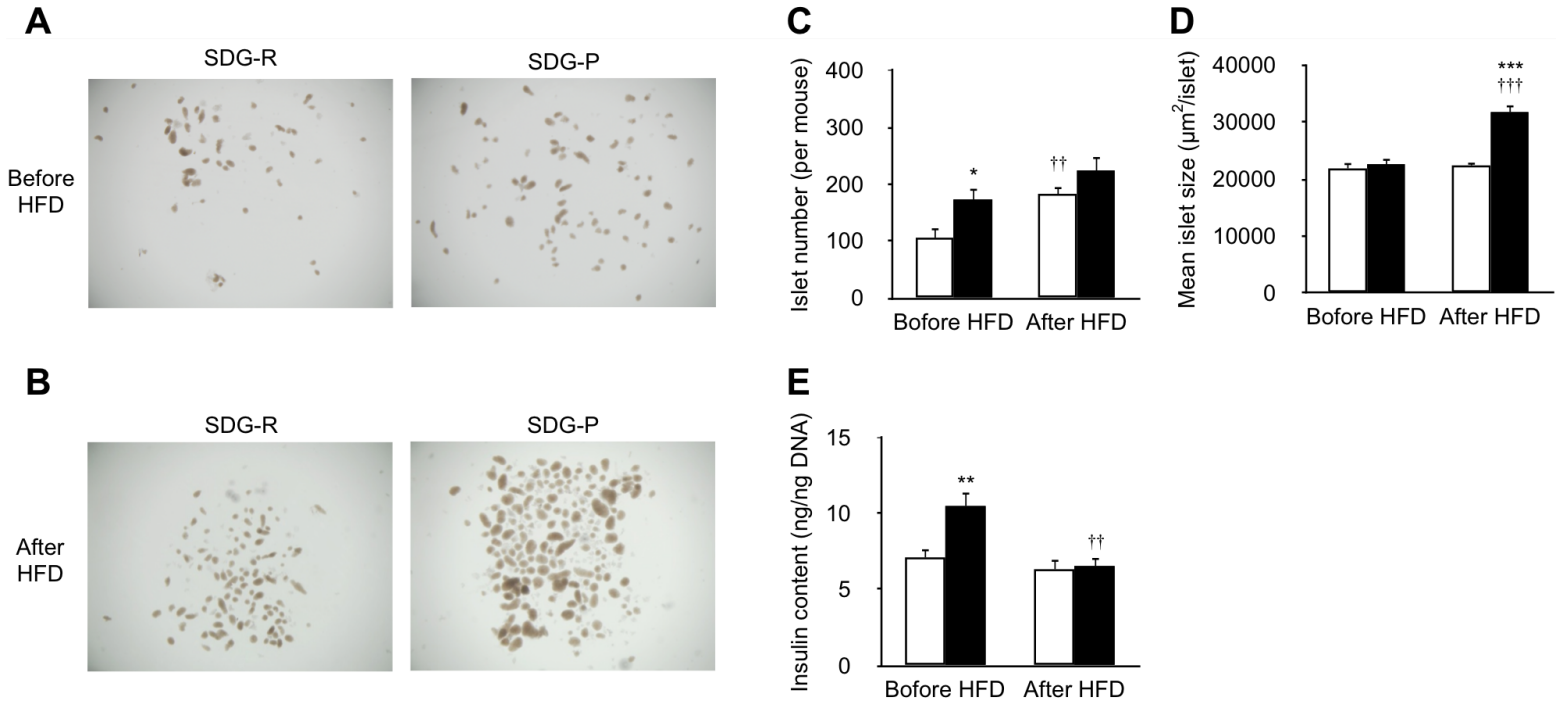
Comparative analyses of isolated islets from SDG-R and SDG-P mice. Stereomicroscopic images of the islets isolated before (**A**) and after 5-week HFD feeding (**B**). Islet numbers (**C**), apparent mean islet size (**D**), and insulin content (**E**) in the isolated islets. Mean ± SEM (n = 9–10). SDG-R, open column; SDG-P, closed column. *p<0.05, **p<0.01, ***p<0.001, *versus* SDG-R mice. †*p*<0.05, ††*p*<0.01, †††*p*<0.01, *versus* before HFD feeding in the same line of mice.

In accordance with the immunohistochemical analysis, the appearance of isolated islets were closely similar between the 2 lines before HFD feeding (Figure 4A, D), whereas enlarged islets were observed in SDG-P mice after receiving HFD (Figure 4B, D). A greater number of islets were harvested from SDG-P mice before HFD feeding; however, no difference in the number of islets after receiving HFD was observed between the 2 lines (Figure 4C). Intracellular insulin content in SDG-P islets was higher than that in SDG-R islets before HFD feeding (Figure 4E). However, after receiving HFD, no difference in insulin content was observed between the 2 lines (Figure 4E). During the 5-week HFD feeding, the total number of islets was increased in SDG-R mice, whereas the average size of islets was increased but the cellular insulin content was decreased in SDG-P mice (Figure 4C–E).

### Insulin Secretion from Isolated Islets

**Figure 5:**
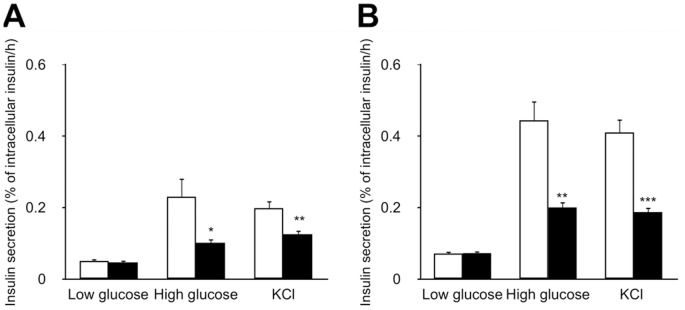
Insulin secretion from isolated islets of SDG-R and SDG-P mice. GSIS and KSIS from the islets isolated before (**A**) and after 5-week HFD feeding (**B**). Insulin secretion in 60 min was normalized to intracellular insulin content and expressed as percentages. Mean ± SEM (n = 9–10). SDG-R, open column; SDG-P, closed column. *p<0.05, **p<0.01, ***p<0.001, *versus* SDG-R mice.

In accordance with the difference in post-glucose challenge insulin response in vivo (Figure 2), the glucose-induced insulin secretion (GSIS) of the isolated islets from SDG-P mice were significantly lower than those from SDG-R mice regardless of whether it was before or after HFD feeding (Figure 5). A similar trend in insulin response was also observed in the KCl-induced insulin secretion (KSIS) (Figure 5).

### Gene Expression in Islets

**Figure 6:**
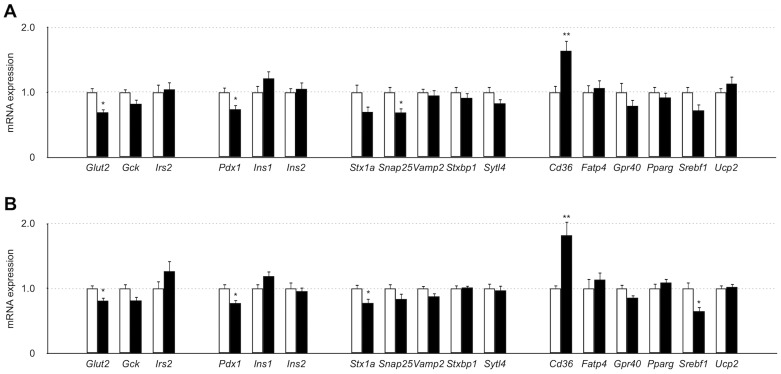
Gene expression levels in isolated islets of SDG-R and SDG-P mice. Relative gene expression levels in the islets isolated before (**A**) and after 5-week HFD feeding (**B**). Gene expression levels were normalized to *Gapdh*, and the normalized expression levels in SDG-P mice (closed column) were expressed as relative values to those in SDG-R mice (open column). Mean ± SEM (n = 7–9). *p<0.05, *versus* SDG-R mice.

The gene expression levels of a glucose transporter, *Glut2*, and a pancreas-specific transcriptional factor, *Pdx1*, were significantly lower in SDG-P islets than in SDG-R islets, regardless of whether it was before or after HFD feeding (Figure 6). The expression levels of the soluble *N*-ethylmaleimide-sensitive factor attachment protein receptor (SNARE) proteins *Snap25* and *Stx1a* in SDG-P islets were significantly lower than those in SDG-R before and after HFD feeding (Figure 6). SNARE proteins play a central role in insulin granule exocytosis in β cells [17]. Among lipid-handling and metabolism-related genes, a fatty acid transporter, *Cd36*, had a higher expression level in SDG-P islets, regardless of whether it was before or after HFD feeding, whereas a transcriptional factor, *Srebf1*, had a lower expression level after HFD feeding in SDG-P mice than in SDG-R mice (Figure 6).

## Discussion

Selective breeding has been applied to develop several animal models for diabetes research [2], [18], [19]. The polygenic background of selectively bred animal models is most likely to mimic the human pathophysiological features of type 2 diabetes. However, existing selectively bred diabetic models display marked hyperglycemia owing to spontaneous β cell loss even on normal chow feeding [10]. Thus, hereditary predisposition to diet-induced impairment of glucose metabolism and consequent development of type 2 diabetes cannot be explained by the existing models. In the present study, SDG-P mice showed impaired glucose tolerance (moderate hyperglycemia in the postchallenge, not fasting, condition) before HFD feeding and developed overt diabetes after receiving HFD. Meanwhile, SDG-R mice maintained normal glucose tolerance even after receiving HFD. These novel mouse lines will therefore be appropriate to investigate the predisposition to diet-induced diabetes.

Before receiving HFD, SDG-P mice already displayed higher postchallenge blood glucose levels than SDG-R mice. In addition, the postchallenge insulin response was significantly lower in SDG-P mice than in SDG-R mice. These results suggest that SDG-P mice had innate defects in insulin secretion of pancreatic β cells. Several epidemiological studies also demonstrated that low early-phase insulin response in OGTT can predict future development of type 2 diabetes [20]–[22]. The preceding impairment of insulin secretion may predispose to exacerbated glucose tolerance under excessive dietary fat intake.

To elucidate the determinants of the difference in postchallenge insulin response between SDG-R and SDG-P mice, we included an analysis of the morphological features and functions of the pancreatic islets. Before HFD feeding, a greater number of islets with higher insulin content were harvested from SDG-P mice as compared with SDG-R mice, whereas no apparent differences were observed on immunohistological examination between the 2 lines, suggesting that functional defect (impaired insulin secretion) rather than β cell mass determined the impaired postchallenge insulin response in SDG-P mice in vivo. As expected, the isolated islets from SDG-P mice showed lower GSIS in vitro even before receiving HFD. The feature of impaired insulin secretion in the islets of prediabetic SDG-P mice resembled that of diabetic GK rats [23] and NSY mice [18].

Prevailing rodent models for type 2 diabetes (*e.g.*, GK rats and db/db mice) exhibit a progressive decline in β cell mass owing to the induction of apoptosis or dedifferentiation [24]–[27]. Several reports suggest that oxidative stress and endoplasmic reticulum stress are related to the β cell loss in these animals [14], [26], [28]. In contrast to the advanced diabetic animals, SDG-P mice showed increased β cell mass with preserved structural integrity of islets after HFD feeding. In addition, we could not observe increased apoptotic cell death in SDG-P islets even after HFD feeding (by TUNEL staining, data not shown). However, longer-term HFD feeding than the present study may eventually lead to apoptotic β cell death in SDG-P mice by excessive metabolic stress.

The gene expression patterns in SDG-P islets were notably almost unchanged (relative to SDG-R islets) after receiving HFD, implying that the β cell dysfunction (*i.e.*, impaired insulin secretion) is a hereditary character, not an acquired one. During HFD feeding, the islet size was increased in SDG-P mice, most likely due to the β cell proliferation as a compensatory response to hyperglycemia [29], [30]. However, the β cell adaptation only in mass, but not in function, was insufficient to ameliorate the glucose intolerance in SDG-P mice. In accordance with the results of islet studies of GK rats and patients with type 2 diabetes [31], reduced gene expression levels of *Glut2* and SNARE proteins were observed in SDG-P islets, suggesting impairments in glucose uptake and exocytosis machinery of the insulin granules in β cells. Accordingly, GSIS and KSIS were impaired in SDG-P islets. A reduced gene expression level of *Pdx1*, a master regulator of β cell proliferation and function, may contribute to quantitative and qualitative defects in SDG-P islets.

In addition, higher *Cd36* expression level in SDG-P islets may participate in the acceleration of glucose intolerance under HFD feeding because CD36 protein (also known as fatty acid translocase) is postulated to facilitate fatty acid uptake, which leads to the attenuation of GSIS in β cells [32], [33]. Chronic exposure to free fatty acids [34]–[36], as well as chronic hyperglycemia [37], [38], is reported to reduce insulin content in pancreatic β cells. In this study, intracellular insulin content was decreased in SDG-P islets by HFD feeding. Thus, the possible involvement of CD36 in attenuated GSIS and decreased insulin content in islets is of further interest.

In conclusion, the present results indicate that the HFD-induced glucose intolerance-prone (SDG-P) mice had a hereditary defect in insulin secretion as compared with the glucose intolerance-resistant (SDG-R) mice. Lower gene expression levels involved in glucose uptake and insulin granule exocytosis may contribute to defects in SDG-P islets. The innate predisposition in pancreatic islets may determine the susceptibility to diet-induced acceleration of glucose intolerance. Recently, we have reported SDG-P mice showed 4-fold greater atherosclerotic lesion formation than SDG-R mice on an atherogenic diet [39], indicating that these mice may also serve as useful in vivo models for investigating the causal role of glucose intolerance in the pathogenesis of atherosclerosis. Further studies with these novel polygenic model mice are warranted to provide new strategies for the prevention and treatment of diet-induced type 2 diabetes and its complications.
